# Awareness of colorectal cancer signs and symptoms: a national cross-sectional study from Palestine

**DOI:** 10.1186/s12889-022-13285-8

**Published:** 2022-04-30

**Authors:** Mohamedraed Elshami, Mohammed Ayyad, Mohammed Alser, Ibrahim Al-Slaibi, Shoruq Ahmed Naji, Balqees Mustafa Mohamad, Wejdan Sudki Isleem, Adela Shurrab, Bashar Yaghi, Yahya Ayyash Qabaja, Fatima Khader Hmdan, Mohammad Fuad Dwikat, Raneen Raed Sweity, Remah Tayseer Jneed, Khayria Ali Assaf, Maram Elena Albandak, Mohammed Madhat Hmaid, Iyas Imad Awwad, Belal Khalil Alhabil, Marah Naser Alarda, Amani Saleh Alsattari, Moumen Sameer Aboyousef, Omar Abdallah Aljbour, Rinad AlSharif, Christy Teddy Giacaman, Ali Younis Alnaga, Ranin Mufid Abu Nemer, Nada Mahmoud Almadhoun, Sondos Mahmoud Skaik, Nasser Abu-El-Noor, Bettina Bottcher

**Affiliations:** 1 Division of Surgical Oncology, Department of Surgery, University Hospitals Cleveland Medical Center, 11100 Euclid Avenue, Lakeside 7100, Cleveland, OH USA; 2 Ministry of Health, Gaza, Palestine; 3 Faculty of Medicine, Al-Quds University, Jerusalem, Palestine; 4 Almakassed Hospital, Jerusalem, Palestine; 5 Faculty of Pharmacy, Al-Azhar University of Gaza, Gaza, Palestine; 6 Beit Jala Governmental Hospital (Al-Hussein), Bethlehem, Palestine; 7 Faculty of Medicine, Islamic University of Gaza, Gaza, Palestine; 8 Palestine Medical Complex, Khanyounis, Palestine; 9 Faculty of Medicine, An-Najah National University, Nablus, Palestine; 10 Faculty of Dentistry, Arab American University, Jenin, Palestine; 11 Faculty of Nursing and Health Sciences, Bethlehem University, Bethlehem, Palestine; 12 Faculty of Allied Medical Sciences, Arab American University, Jenin, Palestine; 13 Faculty of Medicine, Al-Azhar University, Gaza, Palestine; 14 Faculty of Medicine, Al-Quds Abu Dis University Al-Azhar Branch of Gaza, Gaza, Palestine; 15 Faculty of Nursing, Islamic University of Gaza, Gaza, Palestine

**Keywords:** Colorectal cancer, Awareness, Signs, Symptoms, Early detection, Early presentation, Health education, Palestine

## Abstract

**Background:**

In low-resource settings, the awareness level of colorectal cancer (CRC) signs and symptoms plays a crucial role in early detection and treatment. This study examined the public awareness level of CRC signs and symptoms in Palestine and investigated the factors associated with good awareness.

**Methods:**

This was a national cross-sectional study conducted at hospitals, primary healthcare centers, and public spaces in 11 governorates across Palestine between July 2019 and March 2020. A translated-into-Arabic version of the validated bowel cancer awareness measure (BoCAM) was utilized to assess the awareness level of CRC signs and symptoms. For each correctly identified CRC sign/symptom, one point was given. The total score (ranging from 0 to 12) was calculated and categorized into three categories based on the number of symptoms recognized: poor (0 to 4), fair (5 to 8), and good awareness (9 to 12).

**Results:**

Of 5254 approached, 4877 participants completed the questionnaire (response rate = 92.3%). A total of 4623 questionnaires were included in the analysis; 1923 were from the Gaza Strip and 2700 from the West Bank and Jerusalem (WBJ). Participants from the Gaza Strip were younger, gained lower monthly income, and had less chronic diseases than participants in the WBJ.

The most frequently identified CRC sign/symptom was ‘lump in the abdomen’ while the least was ‘pain in the back passage’. Only 1849 participants (40.0%, 95% CI: 39.0%-41.0%) had a good awareness level of CRC signs/symptoms. Participants living in the WBJ were more likely to have good awareness than participants living in the Gaza Strip (42.2% vs. 37.0%; *p* = 0.002). Knowing someone with cancer (OR = 1.37, 95% CI: 1.21–1.55; *p* < 0.001) and visiting hospitals (OR = 1.46, 95% CI: 1.25–1.70; *p* < 0.001) were both associated with higher likelihood of having good awareness. However, male gender (OR = 0.80, 95% CI: 0.68–0.94; *p* = 0.006) and following a vegetarian diet (OR = 0.59, 95% CI: 0.48–0.73; *p* < 0.001) were both associated with lower likelihood of having good awareness.

**Conclusion:**

Less than half of the study participants had a good awareness level of CRC signs and symptoms. Future education interventions are needed to improve public awareness of CRC in Palestine.

**Supplementary Information:**

The online version contains supplementary material available at 10.1186/s12889-022-13285-8.

## Introduction

Colorectal cancer (CRC) is the third most common cancer and the second common cause of cancer-related mortality globally with 1,931,590 new cases and 935,173 deaths in 2020 [1]. In Palestine, CRC is the second most common cancer in both males and females with incidence rates of 15.2 per 100,000 general population in the West Bank and Jerusalem (WBJ) and 11.5 per 100,000 general population in the Gaza Strip [2, 3]. In 2020, cancer was the third most common cause of mortality in Palestine constituting 14.1% of total reported deaths. CRC was the second highest cause of death among all cancers making up 13.9% of all cancer-related deaths in Palestine [4].

The global burden of cancer is predicted to rise by 47.0% in 2040 compared to 2020 [5]. This is anticipated to affect low- and middle-income countries (LMICs), like Palestine, to a significantly greater extent than high-income countries (HICs) with predicted increases of 64.0% to 95.0% and 32.0% to 56.0% respectively [5]. Simultaneously, the associated mortality is predicted to rise in LMICs significantly, while cancer-associated mortality in HICs is predicted to follow its current trend of remaining stable or decreasing [6]. An estimate of 75.0% of the global cancer mortality is predicted to occur in LMICs by 2030 [7]. Factors contributing to these disparate trends are many, including lack of screening programs in LMICs, poorer risk factor control, improved cancer therapies in HICs, lack of educational resources in LMICs and prolonged times to diagnosis in LMICs [8, 9]. In Palestine, too, no screening program exists for CRC, access to treatment is often difficult and cancer-related mortality is high and often judged avoidable [10, 11]. In order to improve outcomes from cancer treatment, the interval from first signs and symptoms to diagnosis has to be shortened.

Signs and symptoms like abdominal and rectal masses, iron deficiency anemia, rectal bleeding, and change in bowel habits could be suggestive of CRC [12]. Good recognition of these signs and symptoms may facilitate early presentation, which increases the chances of patients to be diagnosed in early stages and have higher survival rates [13–15].

A previous study conducted in the Gaza Strip showed poor awareness of CRC signs and symptoms; highlighting the need to explore the level of national awareness about CRC in Palestine [16]. This is especially important given that there is no established screening program for CRC in Palestine [17]. High awareness of CRC signs and symptoms may enhance early diagnosis, which could potentially reduce the socioeconomic and health burden of CRC in Palestine.

This national study aimed to: (i) evaluate the Palestinians’ awareness level of CRC signs and symptoms, (ii) compare CRC awareness in the two main areas of Palestine; the Gaza Strip vs. the WBJ, and (iii) explore the factors associated with good awareness of CRC signs and symptoms.

## Materials and methods

### Study design and population

This was a national cross-sectional study. It was conducted from the 16^th^ of July 2019 to the 31^st^ of March 2020. The target population was adult Palestinians living in the Gaza Strip or the WBJ. Palestine consists of 16 governorates: five located in the Gaza Strip, and 11 in the WBJ. Participants were recruited from 11 governorates from all over Palestine: four in the Gaza Strip, and seven in the WBJ [18].

### Sampling methods and data collection

The Palestinian Ministry of Health (MoH) has 11 general hospitals with a bed capacity of ≥ 100: six in the West Bank and five in the Gaza Strip [17]. There is no MoH hospital in Jerusalem. However, there are two hospitals with a bed capacity of ≥ 100 owned by non-governmental organizations. There are 26 MoH PHCs that provide all primary healthcare services (i.e., classified as level four): 17 are in the WBJ and nine in the Gaza Strip [17]. Public spaces at the governorates of the corresponding hospitals and PHCs were also targeted. Those included markets, mosques, churches, public transportations, neighborhoods, malls, gardens, and others. Stratified convenience sampling was used to recruit study participants in concordance with previous studies [16, 19–24]. Potential participants could be either visitors in waiting rooms at hospitals and PHCs or visitors to public spaces in 11 out of 16 governorates of Palestine (four in the Gaza Strip and seven in the WBJ). Data were collected on a daily basis by the team of authors who were all working or studying in a health-related field and had been trained on how to approach potential participants, explain the purpose of the study and gain informed consent. Participants were invited to complete the questionnaire in face-to-face interviews at the time of recruitment. Data were collected utilizing ‘Kobo Toolbox’, a secure, user-friendly data collection tool that can be accessed via smartphones [25]. The average time to complete the questionnaire was about seven minutes.

### Inclusion and exclusion criteria

Inclusion criteria included being an adult Palestinian (≥ 18 years), being a visitor in one of the data collection sites as well as the ability and willingness to provide an informed consent to participate in the study. Exclusion Criteria included being a visitor to the oncology departments, working or studying in the medical field, holding a nationality other than Palestinian, and being unable to complete the questionnaire.

### Questionnaire

Data were collected utilizing a modified and translated-into-Arabic version of the Bowel Cancer Awareness Measure (BoCAM) [26]. The questionnaire was translated from English to Arabic for the purpose of this study and then was back translated into English. Each step was done by two different bilingual healthcare professionals with expertise in clinical research and survey design, some of whom were part of the research team, others were researchers at local universities. To ensure content validity, the questionnaire was then reviewed by five independent experts in the fields of gastroenterology, coloproctology, and public health. This was followed by conducting a pilot study (*n* = 25) to assess the clarity of the questions in the Arabic BoCAM. The questionnaires of the pilot study were not included in the final analysis. Finally, internal consistency was evaluated using Cronbach’s Alpha, which reached an acceptable value (α = 0.887).

The questionnaire consisted of two sections. The first section included socio-demographic questions including age group, gender, educational level, occupation, monthly income, place of residency, marital status, having a chronic disease, following a vegetarian diet, knowing someone with cancer, and site of data collection. The second section comprised 12 questions that assessed the recognition of CRC signs and symptoms using a 5-point Likert scale (1 = strongly disagree, 5 = strongly agree). The questions in the original BoCAM with yes/no/unknown responses were modified into 5-point Likert scale questions. This was intended to minimize the possibility of participants answering questions randomly [22, 26, 27]. The signs/symptoms of ‘unexplained generalized fatigue’, ‘unexplained loss of appetite’, and ‘feeling persistently full’ were added to the questionnaire since they were mentioned in other forms of the Cancer Awareness Measure [28–30], and it was thought that it would be helpful to include them in the context of CRC.

### Outcomes

The primary outcome measure was the level of public awareness about CRC signs and symptoms. Secondary outcomes included the proportion of people recognizing each CRC sign and symptom.

### Statistical analysis

The latest recommendation of the American Cancer Society for people at average risk of CRC is to start screening at the age of 45 [31]. Therefore, participants’ age was categorized into two categories using this cutoff: 18–44 years and ≥ 45 years. The monthly income was also categorized into two categories: < 1450 NIS and ≥ 1450 NIS. The cutoff of 1450 NIS (about US$450) was used as it is the minimum wage in Palestine [32].

Continuous variables were described using the median and interquartile range (IQR) as they were non-normally distributed. Categorical variables were described using frequencies and percentages. Baseline characteristics of the participants recruited from the WBJ vs. those recruited from the Gaza Strip were compared using Kruskal–Wallis test if the characteristic was continuous or using Pearson's Chi-square test if it was categorical.

The recognition of each CRC sign/symptom was evaluated using a question based on a 5-point Likert scale with ‘strongly agree’ or ‘agree’ as a correct answer, and ‘strongly disagree’, ‘disagree’, or ‘not sure’ as an incorrect answer. CRC signs/symptoms were further categorized into three categories: (i) signs/symptoms with mass or blood, (ii) signs/symptoms of a non-specific nature, and (iii) other gastrointestinal signs/symptoms. Recognition of CRC signs and symptoms was described using frequencies and percentages with comparisons made by Pearson's Chi-Square test. This was followed by running bivariable and multivariable logistic regression analyses. The model of the multivariable analysis included age group, gender, educational level, occupation, monthly income, place of residency, marital status, having a chronic disease, following a vegetarian diet, knowing someone with cancer, and site of data collection. This model was determined a priori based on previous studies [16, 33–39]. Results of the bivariable analyses are provided in additional file 1.

To assess the participants’ awareness level of CRC signs and symptoms, a scoring system was used. Similar scoring systems were also used in previous studies [19, 21–24]. For each correctly recognized CRC sign/symptom, one point was given. The total score (ranging from 0 to 12) was calculated and categorized based on the number of CRC signs and symptoms recognized into three categories: poor (0 to 4), fair (5 to 8), and good awareness (9 to 12). The awareness level of participants recruited from the Gaza Strip was compared with the awareness level of participants recruited from the WBJ using Pearson's Chi-Square test. Bivariable and multivariable logistic regression analyses were also performed to test the association between participant characteristics and having good awareness level.

Complete case analysis was used to handle missing data, which occurred completely at random. Data were analyzed using Stata software version 16.0 (StataCorp, College Station, Texas, United States).

## Results

### Participant characteristics

Of 5254 approached, 4877 participants completed the questionnaire (response rate = 92.3%).

A total of 4623 questionnaires were included in the analysis and 254 were excluded (44 did not meet the inclusion criteria and 210 had missing data). Of those included, 1923 (41.6%) were from the Gaza Strip and 2700 (58.4%) from the WBJ. The median age (IQR) of all participants was 31.0 years (24.0, 43.0) and 1879 (40.6%) were males (Table 1). Participants from the Gaza Strip were younger, gained lower monthly income, had less chronic diseases, and more frequently followed a vegetarian diet than participants from the WBJ.

**Table 1:** Characteristics of study participants

Characteristic	Total (*n* = 4623)	Gaza Strip (*n* = 1923)	WBJ (*n* = 2700)	p-value
**Age**, median [IQR]	31.0 [24.0, 43.0]	30.0 [24.0, 40.0]	32.0 [(24.0, 44.0])	< 0.001
**Age group**, n (%)				
18 to 44	3608 (78.0)	1579 (82.1)	2029 (75.1)	< 0.001
45 or older	1015 (22.0)	344 (17.9)	671 (24.9)	
**Gender**, n (%)				
Female	2744 (59.4)	1213 (63.1)	1531 (56.7)	< 0.001
Male	1879 (40.6)	710 (36.9)	1169 (43.3)	
**Educational level**, n (%)				
Illiterate	65 (1.4)	24 (1.2)	41 (1.5)	< 0.001
Primary	202 (4.4)	72 (3.7)	130 (4.8)	
Preparatory	529 (11.4)	226 (11.8)	303 (11.2)	
Secondary	1421 (30.7)	624 (32.4)	797 (29.5)	
Diploma	460 (10.0)	252 (13.1)	208 (7.7)	
Bachelor	1808 (39.1)	683 (35.5)	1125 (41.7)	
Postgraduate	138 (3.0)	42 (2.3)	96 (3.6)	
**Occupation**, n (%)				
Unemployed	2067 (44.7)	1112 (57.8)	955 (35.4)	< 0.001
Employed	1898 (41.1)	563 (29.3)	1335 (49.4)	
Retired	96 (2.1)	29 (1.5)	67 (2.5)	
Student	562 (12.1)	219 (11.4)	343 (12.7)	
**Monthly income ≥ 1450 NIS**, n (%)	3039 (65.7)	559 (29.1)	2480 (91.9)	< 0.001
**Having a chronic disease**, n (%)	906 (19.6)	314 (16.3)	592 (21.9)	< 0.001
**Following a vegetarian diet**, n (%)	560 (12.1)	386 (20.1)	174 (6.4)	< 0.001
**Knowing someone with cancer**, n (%)	2395 (51.8)	1007 (52.4)	1388 (51.4)	0.52
**Marital status**, n (%)				
Single	1414 (30.6)	548 (28.5)	866 (32.1)	< 0.001
Married	3067 (66.3)	1316 (68.4)	1751 (64.9)	
Divorced	45 (1.0)	28 (1.5)	17 (0.6)	
Widowed	97 (2.1)	31 (1.6)	66 (2.4)	
**Site of data collection**				
Public spaces, n (%)	1450 (31.4)	491 (25.5)	959 (35.5)	< 0.001
Hospitals, n (%)	1659 (35.9)	690 (35.9)	969 (35.9)	
Primary healthcare centers, n (%)	1514 (32.7)	742 (38.6)	772 (28.6)	

*n* number of participants, *IQR* interquartile range, *WBJ* West Bank and Jerusalem

### Good awareness and its associated factors

A total of 1849 participants (40.0%) demonstrated good awareness of CRC signs and symptoms (Table 2). Participants from the WBJ were more likely than participants from the Gaza Strip to have a good awareness level about CRC symptoms (42.2.0% vs. 37.0%).

**Table 2:** Awareness levels of colorectal cancer signs/symptoms among study participants

Level	Total (*n* = 4623) n (%)	Gaza Strip (*n* = 1923) n (%)	WBJ (*n* = 2700) n (%)	*p*-value
Poor	833 (18.0)	365 (19.0)	468 (17.3)	0.002
Fair	1941 (42.0)	847 (44.0)	1094 (40.5)	
Good	1849 (40.0)	711 (37.0)	1138 (42.2)	

*n* number of participants, *WBJ* West Bank and Jerusalem

On the multivariable analysis, knowing someone with cancer (OR = 1.37, 95% CI: 1.21–1.55; *p* < 0.001) and visiting hospitals (OR = 1.46, 95% CI: 1.25–1.70; *p* < 0.001) were both associated with higher likelihood of having good awareness of CRC signs and symptoms (Table 3). However, male gender (OR = 0.80, 95% CI: 0.68–0.94; *p* = 0.006) and following a vegetarian diet (OR = 0.59, 95% CI: 0.48–0.73; *p* < 0.001) were associated with a lower likelihood of having good awareness.

**Table 3:** Association between having a good awareness of colorectal cancer signs/symptoms and participant characteristics

Characteristic	Good awareness			
	**COR (95% CI)**	***p*-value**	**AOR (95% CI)**^**a**^	***p*-value**
**Age group**				
18 to 44	Ref	Ref	Ref	Ref
45 or older	1.18 (1.02- 1.35)	0.024	1.08 (0.90- 1.28)	0.41
**Gender**				
Female	Ref	Ref	Ref	Ref
Male	0.95 (0.85- 1.08)	0.44	0.80 (0.68- 0.94)	0.006
**Educational level**				
Illiterate	Ref	Ref	Ref	Ref
Primary	1.74 (0.98- 3.09)	0.06	1.92 (0.99- 3.44)	0.06
Preparatory	0.93 (0.55- 1.59)	0.80	1.08 (0.63- 1.87)	0.77
Secondary	1.08 (0.64- 1.80)	0.78	1.32 (0.78- 2.25)	0.30
Diploma	1.12 (0.65- 1.91)	0.68	1.47 (0.84- 2.58)	0.17
Bachelor	1.19 (0.72- 1.99)	0.50	1.57 (0.91- 2.68)	0.10
Postgraduate	1.35 (0.74- 2.48)	0.33	1.72 (0.91- 3.23)	0.09
**Occupation**				
Unemployed	Ref	Ref	Ref	Ref
Employed	1.09 (0.96- 1.24)	0.16	1.05 (0.88- 1.24)	0.61
Retired	0.86 (0.56- 1.32)	0.49	0.80 (0.50- 1.27)	0.34
Student	0.76 (0.62- 0.92)	0.005	0.81 (0.64- 1.02)	0.08
**Monthly income**				
< 1450 NIS	Ref	Ref	Ref	Ref
≥ 1450 NIS	1.19 (1.05- 1.34)	0.007	1.00 (0.88- 1.24)	0.97
**Having a chronic disease**				
No	Ref	Ref	Ref	Ref
Yes	1.07 (0.93- 1.25)	0.34	0.98 (0.83- 1.17)	0.85
**Following a vegetarian diet**				
No	Ref	Ref	Ref	Ref
Yes	0.51 (0.42- 0.63)	< 0.001	0.59 (0.48- 0.73)	< 0.001
**Knowing someone with cancer**				
No	Ref	Ref	Ref	Ref
Yes	1.38 (1.23- 1.55)	< 0.001	1.37 (1.21- 1.55)	< 0.001
**Marital status**				
Single	Ref	Ref	Ref	Ref
Married	1.22 (1.07- 1.39)	0.003	1.14 (0.97- 1.34)	0.12
Divorced	0.86 (0.46- 1.62)	0.64	0.82 (0.43- 1.58)	0.56
Widowed	1.49 (0.99- 2.26)	0.06	1.17 (0.74- 1.84)	0.51
**Residency**				
Gaza Strip	Ref	Ref	Ref	Ref
WBJ	1.24 (1.10- 1.40)	< 0.001	1.15 (0.97- 1.36)	0.10
**Site of data collection**				
Public spaces	Ref	Ref	Ref	Ref
Hospitals	1.44 (1.25- 1.67)	< 0.001	1.46 (1.25- 1.70)	< 0.001
Primary healthcare centers	0.89 (0.77- 1.04)	0.13	0.85 (0.72- 1.01)	0.051

*COR* crude odds ratio, *AOR* adjusted odds ratio, *CI* confidence interval, *WBJ* West Bank and Jerusalem

^a^Adjusted for age-group, gender, educational level, occupation, monthly income, having a chronic disease, following a vegetarian diet, knowing someone with cancer, marital status, residency, and site of data collection

### Recognition of CRC signs and symptoms

Among all participants, the most frequently identified CRC sign/symptom was ‘lump in the abdomen’ (*n* = 3421, 74.0%) followed by ‘unexplained weight loss’ (*n* = 3297, 71.3%) (Table 4). These signs/symptoms were also the most identified in both the Gaza Strip and the WBJ. The least identified signs/symptoms were ‘pain in the back passage’ (*n* = 2222, 42.1%) and ‘bowel does not completely empty after using the lavatory’ (*n* = 2404, 52.0%). These signs/symptoms were also the least identified in both the Gaza Strip and the WBJ.

**Table 4:** Recognition of colorectal cancer signs/symptoms

Category of sign/symptom	Sign/symptom	Total (*n* = 4623) n (%)	Gaza Strip (*n* = 1923) n (%)	WBJ (*n* = 2700) n (%)	p-value
**Signs/symptoms with a mass or blood**	Lump in the abdomen	3421 (74.0)	1505 (78.3)	1916 (71.0)	< 0.001
	Blood in the stools	3119 (67.5)	1275 (66.3)	1844 (68.3)	0.15
	Bleeding from back passage	2753 (59.6)	1220 (63.4)	1533 (56.8)	< 0.001
**Signs/symptoms of a non-specific nature**	Unexplained weight loss	3297 (71.3)	1389 (72.2)	1908 (70.7)	0.25
	Unexplained generalized fatigue	3225 (69.8)	1323 (68.8)	1902 (70.4)	0.23
	Unexplained loss of appetite	2872 (62.1)	1232 (64.1)	1640 (60.7)	0.022
	Anemia	2797 (60.5)	1121 (58.3)	1676 (62.1)	0.010
**Other gastrointestinal signs/symptoms**	Feeling persistently full	2744 (59.4)	1102 (57.3)	1642 (60.8)	0.017
	Change in bowel habits	2686 (58.1)	1013 (52.7)	1673 (62.0)	< 0.001
	Persistent pain in the abdomen	2674 (57.8)	1042 (54.2)	1632 (60.4)	< 0.001
	Bowel does not completely empty after using the lavatory	2404 (52.0)	906 (47.1)	1498 (55.5)	< 0.001
	Pain in the back passage	2222 (42.1)	864 (44.9)	1358 (50.3)	< 0.001

*n* number of participants, *WBJ* West Bank and Jerusalem

### Association between recognizing signs/symptoms with mass or blood and participant characteristics

On the multivariable analysis, participants residing in the WBJ were less likely than participants residing in the Gaza Strip to recognize ‘lump in the abdomen’ (OR = 0.64, 95% CI: 0.53–0.77) and ‘bleeding from back passage’ (OR = 0.70, 95% CI: 0.59–0.82) as CRC signs/symptoms (Supplementary table 1). In addition, participants who suffered from a chronic disease were less likely than participants who did not have a chronic disease to recognize ‘lump in the abdomen’ (OR = 0.82, 95% CI: 0.68–0.98). Moreover, male participants were less likely than female participants to recognize ‘bleeding from back passage’ (OR = 0.73, 95% CI: 0.62–0.86).

On the other hand, vegetarian participants were more likely than non-vegetarian participants to identify ‘lump in the abdomen’ (OR = 1.43, 95% CI: 1.13–1.80) and ‘bleeding from back passage’ (OR = 1.47, 95% CI: 1.21–1.80). Additionally, participants who knew someone with cancer had a higher likelihood than participants who did not to identify ‘lump in the abdomen’ (OR = 1.22, 95% CI: 1.07–1.40). Participants recruited from hospitals were more likely than participants recruited from public spaces to identify ‘blood in the stools’ (OR = 1.20, 95% CI: 1.02–1.41) and ‘bleeding from back passage’ (OR = 1.17, 95% CI: 1.01–1.36).

### Association between recognizing signs/symptoms of a non-specific nature and participant characteristics

Vegetarians were less likely than non-vegetarians to recognize all CRC signs and symptoms of a non-specific nature (Supplementary table 2). In addition, male participants were less likely than female participants to recognize three out of four CRC signs and symptoms of a non-specific nature. On the contrary, participants who knew someone with cancer and those recruited from hospitals were more likely to recognize all CRC signs and symptoms of a non-specific nature. Participants aged ≥ 45 years had a higher likelihood than younger participants (18–44 years) to recognize ‘anemia’ (OR = 1.34, 95% CI: 1.12–1.61).

### Association between recognizing other gastrointestinal signs/symptoms and participant characteristics

Vegetarians were less likely than non-vegetarians to recognize all other gastrointestinal signs/symptoms except ‘persistent pain in the abdomen’ for which no difference was found (Supplementary table 3). Conversely, participants who knew someone with cancer had a higher likelihood than participants who did not to recognize all other gastrointestinal signs/symptoms. In addition, participants recruited from hospitals were more likely than participants recruited from public spaces to recognize all other gastrointestinal signs/symptoms except ‘pain in the back passage’ for which no difference was found.

## Discussion

Good awareness was exhibited by 40.0% of the study participants. Participants from the WBJ had a higher likelihood of having good awareness than participants from the Gaza Strip. Knowing someone with cancer and visiting hospitals were associated with higher odds of having good awareness. In contrast, male gender and following a vegetarian diet were both associated with lower odds of having good awareness. The most frequently identified CRC sign/symptom was ‘lump in the abdomen’ while the least was ‘pain in the back passage’.

Poor awareness of CRC signs and symptoms has been found to be associated with delayed presentation, which may lead to diagnosis at advanced stages [40–42]. Therefore, raising and sustaining high awareness of CRC signs and symptoms should be prioritized in future public health actions. This is especially important where no screening programs exist, as in Palestine [12].

In concordance with this study, previous studies reported low levels of CRC awareness in other Arab countries including Lebanon (33% displayed good awareness), Jordan (34.5%), Qatar (40.2%), and United Arab Emirates (< 50.0%) [43–46]. Conversely, studies in non-Arab countries showed better CRC awareness: the United States of America (91.0% showed good awareness), the United Kingdom (88.0%) Malaysia (70.9%), Turkey (69.0%), and Norway (60.0%) [47–51]. This may reflect poor health education about CRC signs and symptoms in Arab countries and underline the need for establishing continuous educational programs. Another contributing factor could be the shared, misleading cultural beliefs in these countries [52]. A living example of this was noticed during the data collection for this study. Some people thought that God protects them from getting CRC, which could lead to more negligence about the recognition of possible CRC signs/symptoms and late diagnosis.

A previous study in Denmark showed that cancer was perceived as a terminal illness that cannot be treated and screening was only relevant for symptomatic patients [53]. The hopelessness and helplessness people feel towards CRC could be the result of their poor insight—healthcare illiteracy—towards the role of early detection in the morbidity and mortality of the disease [52]. Thus, a multidisciplinary approach should be considered to raise the public awareness about the importance of early detection. This may include creating a curriculum that provides the public with a comprehensive understanding of CRC [53]. Improved awareness following an educational intervention has been shown to improve the ability to recognize and recall the signs and symptoms of cancer for six months after the intervention took place [54]. A previous study in Malaysia showed that individuals exposed to a mass media advertisement campaign for CRC awareness via radio, television, or print format, were more likely to recognize CRC signs and symptoms with more confidence than those who had not been exposed [55]. This could be a component of an efficient strategy in countries with low-resource settings, such as Palestine, where the public can be targeted using mass media, but also an individualized approach like integrating CRC signs and symptoms can be employed in school and university curricula [54–56].

### Differences in the awareness level between the Gaza Strip vs. the WBJ

In this study, the finding that participants from the WBJ were more likely to have good awareness than participants from the Gaza Strip could be related to several factors. Firstly, the lower availability of healthcare resources in the Gaza Strip might make it more challenging for people to access treating facilities, which may reduce their chance to be exposed to health education activities in these facilities [57]. Secondly, digital access to health education sources (e.g., social media) might be different between the two areas. This is primarily due to the regular power cuts experienced by people living in the Gaza Strip [58]. Finally, there could be a variation in the frequency and location of routine health check-ups and follow-ups in the Gaza Strip vs. the WBJ. The Palestinian MoH reported higher numbers and percentages of visitors to its facilities in the WBJ than in the Gaza Strip [32, 57]. The greater exposure to MoH facilities in the WBJ might have helped the participants living there accumulate more knowledge about health topics including CRC [19–22]. Additionally, with the higher median income in the WBJ [32], people living there may be more able to visit private healthcare centers. This may further support people living in the WBJ to enrich their health literacy.

### Factors associated with good awareness of CRC signs and symptoms

In concordance with previous studies [43, 44], in this study, participants who knew someone with cancer or visited hospitals had an increased likelihood of having good awareness of CRC signs and symptoms compared with those who did not. People exposed to sick relatives and patients are personally more concerned and intimidated by the disease and thus are more likely to seek information about health-related topics including CRC [22]. On the contrary, male gender and following a vegetarian diet were found in this study to be associated with a decrease in the likelihood of displaying good awareness of CRC signs and symptoms. This is in line with previous studies which showed that women were more aware about CRC signs and symptoms overall [16, 21, 37]. It could be that females are more frequently exposed to healthcare professionals than males, due to their maternity care experiences. This is supported with the finding that visiting hospitals was associated with a higher likelihood to have good awareness of CRC signs and symptoms. Furthermore, females more frequently take on the care of sick relatives and, thus, become more familiar with health issues. Participants on a vegetarian diet had a lower likelihood to have a good awareness of CRC signs and symptoms. Vegetarians are usually expected to follow a healthier lifestyle [59], which might drive them to read more about health-related topics including CRC. Interestingly, in this study, vegetarians were less likely to recognize most CRC signs and symptoms. This unexpected finding could be potentially explained by the reason for following a vegetarian diet. While this variable was not captured in this study, there was an association between monthly income and following a vegetarian diet, where participants with a lower wage (< 1450 NIS) were about three times more likely to be on a vegetarian diet (OR = 3.22, 95% CI: 2.64–3.93; data not shown). This suggests that inability to afford meat could be the reason for following a vegetarian diet not the intent to have a healthier lifestyle, which might explain the lower likelihood of vegetarians to recognize most CRC signs and symptoms.

## Future directions

The findings of this study underline the substantial need to establish sustainable educational programs that should focus on raising the public awareness of CRC signs and symptoms. Awareness campaigns should be tailored to be appropriate for the specific cultural needs. Improving the awareness of CRC may make the public feel more confident and encourage them to discuss their symptoms with healthcare professionals as soon as they recognize them. This may facilitate early detection and diagnosis of CRC and may improve patient prognosis.

## Strengths and limitations

The major strengths of this study included the large sample size from different areas in Palestine, the high response rate, and the use of a translated version of the validated BoCAM. In addition, the face-to-face interviews for data collection minimized the possibility that a participant could use the internet to answer questions correctly. On the other hand, limitations of this study included the use of stratified convenience sampling, which does not guarantee creating a representative sample of the pubic in Palestine. Nonetheless, the large sample size, the high response rate, and the data collection from different geographical areas across Palestine and from various locations (i.e., hospitals, PHCs, and public spaces) may mitigate this. Another limitation is the exclusion of visitors to the oncology departments and participants with medical backgrounds, which could possibly reduce the number of participants with a presumably good awareness of CRC signs and symptoms. However, the exclusion of these participants was intended to increase the relevancy of this study as a measure of the public awareness. Finally, the study included participants who did not experience actual CRC symptoms, but looked at their perceived knowledge.

## Conclusions

Only 40.0% of the study participants had good awareness of CRC signs and symptoms. Participants living in the WBJ were more likely to have good awareness than participants living in the Gaza Strip. The most frequently identified CRC symptom was ‘lump in the abdomen’ while the least was ‘pain in the back passage’. Knowing someone with cancer and visiting hospitals were both associated with higher likelihoods of having good awareness. However, male gender and following a vegetarian diet were both associated with lower likelihoods of having good awareness. Future education interventions are needed to improve awareness of CRC signs and symptoms and, thus, may improve early diagnosis and survival of CRC patients in Palestine.

### Supplementary Information

**Additional file 1:** **Table 1.** Multivariable logistic regression analyzing the association between the recognition of colorectal cancer signs/symptoms with mass/blood and participant characteristics. **Table 2.** Multivariable logistic regression analyzing the association between the recognition of colorectal cancer signs/symptoms of a non-specific nature and participant characteristics. **Table 3.** Multivariable logistic regression analyzing the association between the recognition of other gastrointestinal signs/symptoms and participant characteristics. **Table 4.** Bivariable logistic regression analyzing the association between recognizing colorectal cancer symptoms with mass/blood and participant characteristics. **Table 5.** Bivariable logistic regression analyzing the association between recognizing colorectal cancer symptoms of a non-specific nature and participant characteristics. **Table 6.** Bivariable logistic regression analyzing the association between recognizing other gastrointestinal symptoms and participant characteristics.

## Data Availability

The dataset used and analyzed during the current study is available from the corresponding author on reasonable request.

## Abbreviations

CRC: Colorectal cancer
WBJ: West Bank and Jerusalem
LMICs: Low- and middle-income countries
HICs: High-income countries
MoH: Ministry of health
BoCAM: Bowel cancer awareness measure
CI: Confidence interval
OR: Odds ratio
