# Correction: Awareness of colorectal cancer signs and symptoms: a national cross-sectional study from Palestine

**DOI:** 10.1186/s12889-023-16709-1

**Published:** 2023-09-18

**Authors:** Mohamedraed Elshami, Mohammed Ayyad, Mohammed Alser, Ibrahim Al-Slaibi, Shoruq Ahmed Naji, Balqees Mustafa Mohamad, Wejdan Sudki Isleem, Adela Shurrab, Bashar Yaghi, Yahya Ayyash Qabaja, Fatima Khader Hmdan, Mohammad Fuad Dwikat, Raneen Raed Sweity, Remah Tayseer Jneed, Khayria Ali Assaf, Maram Elena Albandak, Mohammed Madhat Hmaid, Iyas Imad Awwad, Belal Khalil Alhabil, Marah Naser Alarda, Amani Saleh Alsattari, Moumen Sameer Aboyousef, Omar Abdallah Aljbour, Rinad AlSharif, Christy Teddy Giacaman, Ali Younis Alnaga, Ranin Mufid Abu Nemer, Nada Mahmoud Almadhoun, Sondos Mahmoud Skaik, Nasser Abu-El-Noor, Bettina Bottcher

**Affiliations:** 1 Division of Surgical Oncology, Department of Surgery, University Hospitals Cleveland Medical Center, 11100 Euclid Avenue, Lakeside 7100, Cleveland, OH USA; 2 Ministry of Health, Gaza, Palestine; 3 Faculty of Medicine, Al-Quds University, Jerusalem, Palestine; 4 Almakassed Hospital, Jerusalem, Palestine; 5 Faculty of Pharmacy, Al-Azhar University of Gaza, Gaza, Palestine; 6 Beit Jala Governmental Hospital (Al-Hussein), Bethlehem, Palestine; 7 Faculty of Medicine, Islamic University of Gaza, Gaza, Palestine; 8 Palestine Medical Complex, Khanyounis, Palestine; 9 Faculty of Medicine, An-Najah National University, Nablus, Palestine; 10 Faculty of Dentistry, Arab American University, Jenin, Palestine; 11 Faculty of Nursing and Health Sciences, Bethlehem University, Bethlehem, Palestine; 12 Faculty of Allied Medical Sciences, Arab American University, Jenin, Palestine; 13 Faculty of Medicine, Al-Azhar University, Gaza, Palestine; 14 Faculty of Medicine, Al-Quds Abu Dis University Al-Azhar Branch of Gaza, Gaza, Palestine; 15 Faculty of Nursing, Islamic University of Gaza, Gaza, Palestine

**Correction: BMC Public Health 22, 866 (2022)**

**https://doi.org/10.1186/s12889-022-13285-8**

The original publication of this article [1] contained an incorrect author name. The incorrect and correct information is listed below. The original article has been updated.

Incorrect: Mohammed Majed Ayyad

Correct: Mohammed Ayyad
