# Supplementary material for: Awareness of colorectal cancer signs and symptoms: a national cross-sectional study from Palestine
Source: BMC Public Health. 2022 Apr 30;22:866. doi: 10.1186/s12889-022-13285-8 (PMC9063349; doi:10.1186/s12889-022-13285-8)
Supplement: Supplementary file 1 — Additional file 1: Table 1. Multivariable logistic regression analyzing the association between the recognition of colorectal cancer signs/symptoms with mass/blood and participant characteristics. Table 2. Multivariable logistic regression analyzing the association between the recognition of colorectal cancer signs/symptoms of a non-specific nature and participant characteristics. Table 3. Multivariable logistic regression analyzing the association between the recognition of other gastrointestinal signs/symptoms and participant characteristics. Table 4. Bivariable logistic regression analyzing the association between recognizing colorectal cancer symptoms with mass/blood and participant characteristics. Table 5. Bivariable logistic regression analyzing the association between recognizing colorectal cancer symptoms of a non-specific nature and participant characteristics. Table 6. Bivariable logistic regression analyzing the association between recognizing other gastrointestinal symptoms and participant characteristics. [file 12889_2022_13285_MOESM1_ESM.docx]

**Awareness of Colorectal Cancer Signs and Symptoms: A National Cross-sectional Study from Palestine**

Mohamedraed Elshami, MD, MMSc^1,2^*, Mohammed Ayyad^3^*, Mohammed Alser, MD^2^, Ibrahim Al-Slaibi, MD^4^, Shoruq Ahmed Naji^5^, Balqees Mustafa Mohamad, MD^6^, Wejdan Sudki Isleem^7^, Adela Shurrab, MD^8^, Bashar Yaghi^3^, Yahya Ayyash Qabaja^3^, Fatima Khader Hmdan^3^, Mohammad Fuad Dwikat^9^, Raneen Raed Sweity^3^, Remah Tayseer Jneed^7^, Khayria Ali Assaf^9^, Maram Elena Albandak^3^, Mohammed Madhat Hmaid ,MD^7^, Iyas Imad Awwad^3^, Belal Khalil Alhabil^7^, Marah Naser Taher Alarda^10^, Amani Saleh Alsattari ^7^, Moumen Sameer Aboyousef^7^, Omar Abdallah Aljbour^7^, Rinad AlSharif^3^, Christy Teddy Giacaman^11^, Ali Younis Alnaga^7^, Ranin Mufid Abu Nemer^12^, Nada Mahmoud Almadhoun^13^, Sondos Mahmoud Skaik^14^, Nasser Abu-El-Noor, PhD^15#^, Bettina Bottcher, MD, PhD^2#^

*Contributed equally as a first co-author.

^#^Contributed equally as a senior co-author.

^1^Division of Surgical Oncology, Department of Surgery, University Hospitals Cleveland Medical Center, Cleveland, OH, USA.

^2^Ministry of Health, Gaza, Palestine.

^3^Faculty of Medicine, Al-Quds University, Jerusalem, Palestine.

^4^Almakassed Hospital, Jerusalem, Palestine.

^5^Faculty of Pharmacy, Al-Azhar University of Gaza, Gaza, Palestine.

^6^Beit Jala Governmental Hospital (Al-Hussein), Bethlehem, Palestine.

^7^Faculty of Medicine, Islamic University of Gaza, Gaza, Palestine.

^8^Palestine Medical Complex, Khanyounis, Palestine.

^9^Faculty of Medicine, An-Najah National University, Nablus, Palestine.

^10^Faculty of Dentistry, Arab American University, Palestine, Jenin.

^11^Faculty of Nursing and Health Sciences, Bethlehem university, Bethlehem, Palestine.

^12^Faculty of Allied Medical Sciences, Arab American university, Jenin, Palestine.

^13^Faculty of Medicine, Al-Azhar University, Gaza, Palestine.

^14^Faculty of Medicine, Al-Quds Abu Dis University Al-Azhar branch of Gaza, Gaza, Palestine.

^15^Faculty of Nursing, Islamic University of Gaza, Gaza, Palestine.

**Corresponding author**

Mohamedraed Elshami, MD, MMSc

Division of Surgical Oncology

Department of Surgery

University Hospitals Cleveland Medical Center

11100 Euclid Avenue, Lakeside 7100

Cleveland, OH 44106
Phone: 832-245-6055

Email: mohamedraed.elshami@gmail.com

Supplementary table 1: Multivariable logistic regression analyzing the association between the recognition of colorectal cancer signs/symptoms with mass/blood and participant characteristics.

| **Characteristic** | **Lump in the abdomen** | | **Blood in the stools** | | **Bleeding from back passage** | |
| --- | --- | --- | --- | --- | --- | --- |
|  | **AOR (95% CI)*** | **p-value** | **AOR (95% CI)*** | **p-value** | **AOR (95% CI)*** | **p-value** |
| **Age group**  18 to 44  45 or older | Ref  0.92 (0.75- 1.12) | Ref  0.38 | Ref  1.08 (0.90- 1.30) | Ref  0.41 | Ref  1.06 (0.89- 1.27) | Ref  0.49 |
| **Gender**  Female  Male | Ref  0.94 (0.79- 1.12) | Ref  0.51 | Ref  0.88 (0.74- 1.04) | Ref  0.13 | Ref  0.73 (0.62- 0.86) | Ref  <0.001 |
| **Educational level**  Illiterate  Primary  Preparatory  Secondary  Diploma  Bachelor  Postgraduate | Ref  1.85 (1.01- 3.38)  1.38 (0.80- 2.39)  1.77 (1.03- 3.02)  1.64 (0.93- 2.90)  1.59 (0.93- 2.73)  1.41 (0.74- 2.70) | Ref  0.046  0.25  0.037  0.09  0.09  0.30 | Ref  1.67 (0.94- 2.98)  1.19 (0.70- 2.03)  1.68 (1.01- 2.83)  1.90 (1.10- 3.29)  2.09 (1.24- 3.54)  2.52 (1.32- 4.80) | Ref  0.08  0.51  0.048  0.021  0.006  0.005 | Ref  1.47 (0.83- 2.59)  1.12 (0.66- 1.89)  1.40 (0.84- 2.34)  1.55 (0.90- 2.66)  1.68 (0.99- 2.82)  1.57 (0.84- 2.90) | Ref  0.19  0.68  0.20  0.11  0.051  0.16 |
| **Occupation**  Unemployed  Employed  Retired  Student | Ref  0.88 (0.72- 1.06)  1.05 (0.63- 1.76)  0.72 (0.56- 0.93) | Ref  0.19  0.85  0.012 | Ref  0.92 (0.77- 1.10)  0.75 (0.47- 1.20)  0.87 (0.68- 1.11) | Ref  0.38  0.24  0.25 | Ref  1.07 (0.90- 1.28)  0.83 (0.53- 1.30)  0.82 (0.65- 1.03) | Ref  0.43  0.41  0.09 |
| **Monthly income**  < 1450 NIS  ≥ 1450 NIS | Ref  1.28 (1.05- 1.56) | Ref  0.013 | Ref  1.34 (1.12- 1.61) | Ref  0.001 | Ref  1.18 (0.99- 1.41) | Ref  0.06 |
| **Having a chronic disease**  No  Yes | Ref  0.82 (0.68- 0.98) | Ref  0.033 | Ref  1.09 (0.91- 1.31) | Ref  0.33 | Ref  1.11 (0.94- 1.32) | Ref  0.22 |
| **Following a vegetarian diet**  No  Yes | Ref  1.43 (1.13- 1.80) | Ref  0.003 | Ref  1.20 (0.98- 1.47) | Ref  0.08 | Ref  1.47 (1.21- 1.80) | Ref  <0.001 |
| **Knowing someone with cancer**  No  Yes | Ref  1.22 (1.07- 1.40) | Ref  0.004 | Ref  1.07 (0.94- 1.21) | Ref  0.31 | Ref  0.98 (0.87- 1.11) | Ref  0.75 |
| **Marital status**  Single  Married  Divorced  Widowed | Ref  1.27 (1.07- 1.52)  1.38 (0.69- 2.86)  1.13 (0.69- 1.86) | Ref  0.008  0.38  0.62 | Ref  1.16 (0.99- 1.38)  1.03 (0.54- 1.95)  1.06 (0.65- 1.70) | Ref  0.07  0.93  0.82 | Ref  1.27 (1.08- 1.48)  1.19 (0.63- 2.23)  0.91 (0.58- 1.43) | Ref  0.004  0.59  0.68 |
| **Residency**  Gaza Strip  WBJ | Ref  0.64 (0.53- 0.77) | Ref  <0.001 | Ref  0.93 (0.79- 1.11) | Ref  0.44 | Ref  0.70 (0.59- 0.82) | Ref  <0.001 |
| **Site of data collection**  Public spaces  Hospitals  Primary healthcare centers | Ref  0.94 (0.79- 1.11)  0.92 (0.77- 1.10) | Ref  0.45  0.35 | Ref  1.20 (1.02- 1.41)  0.90 (0.76- 1.06) | Ref  0.024  0.22 | Ref  1.17 (1.01- 1.36)  0.80 (0.68- 0.93) | Ref  0.043  0.005 |

AOR= adjusted odds ratio, CI= confidence interval, WBJ= West Bank and Jerusalem.
* Adjusted for age-group, gender, educational level, occupation, monthly income, having a chronic disease, following a vegetarian diet, knowing someone with cancer, marital status, residency, and site of data collection.

| **Characteristic**  Table 3: Association between recognizing breast symptoms and participant characteristics.  Table 3: Association between recognizing breast symptoms and sociodemographic factors. | **Unexplained weight loss** | | **Unexplained generalized fatigue** | | **Unexplained loss of appetite** | | **Anemia** | |
| --- | --- | --- | --- | --- | --- | --- | --- | --- |
|  | **AOR (95% CI)*** | **p-value** | **AOR (95% CI)*** | **p-value** | **AOR (95% CI)*** | **p-value** | **AOR (95% CI)*** | **p-value** |
| **Age group**  18 to 44  45 or older | Ref  1.06 (0.87- 1.29) | Ref  0.54 | Ref  1.02 (0.84- 1.23) | Ref  0.85 | Ref  0.91 (0.76- 1.09) | Ref  0.32 | Ref  1.34 (1.12- 1.61) | Ref  0.002 |
| **Gender**  Female  Male | Ref  0.74 (0.63- 0.89) | Ref  0.001 | Ref  0.67 (0.56- 0.79) | Ref  <0.001 | Ref  0.72 (0.61- 0.84) | Ref  <0.001 | Ref  0.95 (0.81- 1.11) | Ref  0.52 |
| **Educational level**  Illiterate  Primary  Preparatory  Secondary  Diploma  Bachelor  Postgraduate | Ref  1.91 (1.03- 3.54)  1.38 (0.79- 2.42)  1.60 (0.93- 2.77)  1.85 (1.04- 3.28)  1.68 (0.97- 2.92)  1.71 (0.89- 3.31) | Ref  0.041  0.26  0.09  0.037  0.06  0.11 | Ref  1.33 (0.73- 2.44)  1.23 (0.70- 2.14)  1.32 (0.77- 2.28)  1.36 (0.77- 2.41)  1.51 (0.87- 2.62)  1.60 (0.83- 3.11) | Ref  0.35  0.48  0.32  0.29  0.14  0.16 | Ref  0.98 (0.54- 1.78)  0.89 (0.51- 1.55)  0.93 (0.54- 1.60)  0.90 (0.51- 1.60)  0.98 (0.57- 1.69)  1.19 (0.62- 2.27) | Ref  0.94  0.67  0.79  0.73  0.94  0.61 | Ref  2.28 (1.25- 4.15)  1.31 (0.76- 2.25)  1.22 (0.72- 2.07)  1.22 (0.70- 2.13)  1.39 (0.82- 2.37)  1.14 (0.61- 2.14) | Ref  0.007  0.33  0.46  0.47  0.23  0.68 |
| **Occupation**  Unemployed  Employed  Retired  Student | Ref  1.04 (0.86- 1.26)  0.94 (0.57- 1.53)  0.94 (0.74- 1.21) | Ref  0.67  0.79  0.64 | Ref  1.15 (0.95- 1.39)  0.99 (0.61- 1.61)  0.84 (0.65- 1.07) | Ref  0.14  0.99  0.15 | Ref  0.89 (0.75- 1.07)  0.90 (0.57- 1.42)  0.72 (0.57- 0.91) | Ref  0.21  0.66  0.007 | Ref  1.08 (0.90- 1.28)  0.76 (0.48- 1.22)  0.86 (0.69- 1.09) | Ref  0.41  0.26  0.22 |
| **Monthly income**  < 1450 NIS  ≥ 1450 NIS | Ref  1.03 (0.85- 1.24) | Ref  0.78 | Ref  1.07 (0.89- 1.39) | Ref  0.14 | Ref  1.14 (0.96- 1.36) | Ref  0.14 | Ref  1.14 (0.96- 1.36) | Ref  0.14 |
| **Having a chronic disease**  No  Yes | Ref  0.98 (0.81- 1.18) | Ref  0.83 | Ref  0.98 (0.81- 1.17) | Ref  0.81 | Ref  0.97 (0.81- 1.15) | Ref  0.73 | Ref  1.01 (0.85- 1.21) | Ref  0.89 |
| **Following a vegetarian diet**  No  Yes | Ref  0.72 (0.59- 0.88) | Ref  0.001 | Ref  0.53 (0.43- 0.64) | Ref  <0.001 | Ref  0.66 (0.55- 0.80) | Ref  <0.001 | Ref  0.61 (0.51- 0.74) | Ref  <0.001 |
| **Knowing someone with cancer**  No  Yes | Ref  1.58 (1.39- 1.81) | Ref  <0.001 | Ref  1.55 (1.36- 1.77) | Ref  <0.001 | Ref  1.51 (1.33- 1.70) | Ref  <0.001 | Ref  1.35 (1.19- 1.52) | Ref  <0.001 |
| **Marital status**  Single  Married  Divorced  Widowed | Ref  1.19 (0.99- 1.41)  0.91 (0.47- 1.77)  1.40 (0.81- 2.39) | Ref  0.051  0.79  0.23 | Ref  1.01 (0.85- 1.20)  0.56 (0.30- 1.04)  0.87 (0.53- 1.43) | Ref  0.89  0.07  0.58 | Ref  0.99 (0.84- 1.17)  0.53 (0.29- 0.98)  0.84 (0.53- 1.34) | Ref  0.94  0.043  0.46 | Ref  1.05 (0.89- 1.23)  1.12 (0.59- 2.13)  0.91 (0.56- 1.46) | Ref  0.55  0.72  0.69 |
| **Residency**  Gaza Strip  WBJ | Ref  0.89 (0.75- 1.07) | Ref  0.21 | Ref  0.94 (0.79- 1.12) | Ref  0.49 | Ref  0.78 (0.66- 0.93) | Ref  0.004 | Ref  0.99 (0.84- 1.17) | Ref  0.89 |
| **Site of data collection**  Public spaces  Hospitals  Primary healthcare centers | Ref  1.32 (1.12- 1.55)  1.16 (0.98- 1.38) | Ref  0.001  0.09 | Ref  1.23 (1.04- 1.44)  1.06 (0.89- 1.25) | Ref  0.014  0.52 | Ref  1.43 (1.23- 1.67)  0.98 (0.84- 1.16) | Ref  <0.001  0.85 | Ref  1.82 (1.56- 2.13)  1.17 (0.99- 1.37) | Ref  <0.001  0.06 |

Supplementary table 2: Multivariable logistic regression analyzing the association between the recognition of colorectal cancer signs/symptoms of a non-specific nature and participant characteristics.

AOR= adjusted odds ratio, CI= confidence interval, WBJ= West Bank and Jerusalem.
* Adjusted for age-group, gender, educational level, occupation, monthly income, having a chronic disease, following a vegetarian diet, knowing someone with cancer, marital status, residency, and site of data collection.

| **Characteristic**  Supplementary table 3: Multivariable logistic regression analyzing the association between the recognition of other gastrointestinal signs/symptoms and participant characteristics. | **Feeling persistently full** | | **Change in bowel habits** | | | **Persistent pain in the abdomen** | | | **Bowel does not completely empty** | | | **Pain in the back passage** | |
| --- | --- | --- | --- | --- | --- | --- | --- | --- | --- | --- | --- | --- | --- |
|  | **AOR (95% CI)*** | **p-value** | **AOR (95% CI)*** | **p-value** | **AOR (95% CI)*** | | **p-value** | **AOR (95% CI)*** | | **p-value** | **AOR (95% CI)*** | | **p-value** |
| **Age group**  18 to 44  45 or older | Ref  0.98 (0.82- 1.17) | Ref  0.84 | Ref  1.38 (1.15- 1.64) | Ref  <0.001 | Ref  0.89 (0.75- 1.06) | | Ref  0.20 | Ref  1.13 (0.95- 1.35) | | Ref  0.16 | Ref  1.11 (0.94- 1.32) | | Ref  0.22 |
| **Gender**  Female  Male | Ref  0.72 (0.61- 0.84) | Ref  <0.001 | Ref  0.77 (0.65- 0.90) | Ref  0.001 | Ref  0.93 (0.80- 1.10) | | Ref  0.40 | Ref  0.79 (0.67- 0.92) | | Ref  0.003 | Ref  0.87 (0.75- 1.02) | | Ref  0.09 |
| **Educational level**  Illiterate  Primary  Preparatory  Secondary  Diploma  Bachelor  Postgraduate | Ref  1.79 (1.01- 3.18)  1.69 (0.99- 2.88)  1.55 (0.92- 2.59)  1.50 (0.87- 2.59)  1.86 (1.10- 3.14)  2.10 (1.12- 3.92) | Ref  0.046  0.052  0.10  0.14  0.020  0.020 | Ref  1.66 (0.93- 2.96)  1.23 (0.72- 2.09)  1.32 (0.78- 2.22)  1.46 (0.84- 2.52)  1.63 (0.96- 2.75)  1.43 (0.76- 2.66) | Ref  0.09  0.45  0.30  0.18  0.07  0.26 | Ref  1.50 (0.85- 2.64)  1.32 (0.78- 2.24)  1.64 (0.98- 2.75)  2.12 (1.23- 3.64)  2.15 (1.28- 3.62)  2.55 (1.37- 4.77) | | Ref  0.16  0.30  0.06  0.006  0.004  0.003 | Ref  1.55 (0.87- 2.75)  1.11 (0.65- 1.88)  1.20 (0.71- 2.01)  1.21 (0.70- 2.08)  1.31 (0.77- 2.20)  1.23 (0.66- 2.28) | | Ref  0.14  0.71  0.50  0.49  0.32  0.51 | Ref  1.31 (0.75- 2.31)  1.03 (0.61- 1.73)  1.08 (0.65- 1.81)  1.04 (0.61- 1.78)  1.10 (0.66- 1.85)  1.36 (0.74- 2.51) | | Ref  0.35  0.93  0.76  0.89  0.72  0.33 |
| **Occupation**  Unemployed  Employed  Retired  Student | Ref  1.06 (0.89- 1.27)  0.73 (0.46- 1.14)  0.87 (0.69- 1.10) | Ref  0.49  0.17  0.25 | Ref  1.05 (0.88- 1.25)  1.12 (0.70- 1.79)  1.09 (0.86- 1.37) | Ref  0.58  0.63  0.48 | Ref  0.90 (0.76- 1.06)  0.92 (0.59- 1.46)  0.86 (0.68- 1.08) | | Ref  0.21  0.74  0.20 | Ref  0.97 (0.82- 1.15)  0.81 (0.51- 1.27)  0.88 (0.70- 1.10) | | Ref  0.70  0.35  0.27 | Ref  0.95 (0.80- 1.12)  0.91 (0.58- 1.43)  0.80 (0.63- 1.00) | | Ref  0.53  0.69  0.051 |
| **Monthly income**  < 1450 NIS  ≥ 1450 NIS | Ref  1.04 (0.87- 1.24) | Ref  0.66 | Ref  1.07 (0.90- 1.27) | Ref  0.46 | Ref  1.30 (1.09- 1.54) | | Ref  0.003 | Ref  1.27 (1.07- 1.51) | | Ref  0.006 | Ref  1.16 (0.98- 1.37) | | Ref  0.09 |
| **Having a chronic disease**  No  Yes | Ref  0.97 (0.82- 1.15) | Ref  0.75 | Ref  0.91 (0.77- 1.08) | Ref  0.29 | Ref  1.10 (0.93- 1.30) | | Ref  0.28 | Ref  0.96 (0.81- 1.14) | | Ref  0.65 | Ref  1.04 (0.88- 1.23) | | Ref  0.67 |
| **Following a vegetarian diet**  No  Yes | Ref  0.42 (0.35- 0.51) | Ref  <0.001 | Ref  0.45 (0.37- 0.55) | Ref  <0.001 | Ref  0.85 (0.70- 1.03) | | Ref  0.09 | Ref  0.50 (0.41- 0.61) | | Ref  <0.001 | Ref  0.57 (0.47- 0.69) | | Ref  <0.001 |
| **Knowing someone with cancer**  No  Yes | Ref  1.30 (1.15- 1.47) | Ref  <0.001 | Ref  1.25 (1.11- 1.41) | Ref  <0.001 | Ref  1.21 (1.07- 1.36) | | Ref  0.002 | Ref  1.29 (1.14- 1.45) | | Ref  <0.001 | Ref  1.17 (1.04- 1.32) | | Ref  0.008 |
| **Marital status**  Single  Married  Divorced  Widowed | Ref  1.12 (0.96- 1.32)  1.24 (0.65- 2.34)  0.94 (0.59- 1.49) | Ref  0.15  0.52  0.79 | Ref  1.01 (0.86- 1.19)  0.80 (0.43- 1.48)  1.23 (0.76- 1.99) | Ref  0.89  0.47  0.41 | Ref  0.96 (0.81- 1.12)  0.77 (0.41- 1.42)  1.02 (0.64- 1.61) | | Ref  0.59  0.40  0.93 | Ref  1.11 (0.95- 1.30)  0.84 (0.45- 1.56)  0.86 (0.55- 1.36) | | Ref  0.20  0.58  0.52 | Ref  0.96 (0.82- 1.12)  0.87 (0.47- 1.62)  0.88 (0.56- 1.37) | | Ref  0.59  0.67  0.57 |
| **Residency**  Gaza Strip  WBJ | Ref  1.02 (0.86- 1.20) | Ref  0.84 | Ref  1.22 (1.04- 1.44) | Ref  0.015 | Ref  1.09 (0.92- 1.28) | | Ref  0.32 | Ref  1.10 (0.94- 1.30) | | Ref  0.23 | Ref  1.03 (0.88- 1.21) | | Ref  0.72 |
| **Site of data collection**  Public spaces  Hospitals  Primary healthcare centers | Ref  1.53 (1.31- 1.78)  1.06 (0.90- 1.25) | Ref  <0.001  0.47 | Ref  1.25 (1.07- 1.45)  0.86 (0.73- 1.01) | Ref  0.005  0.07 | Ref  1.34 (1.15- 1.56)  0.87 (0.74- 1.02) | | Ref  <0.001  0.08 | Ref  1.37 (1.18- 1.59)  0.81 (0.69- 0.95) | | Ref  <0.001  0.008 | Ref  1.04 (0.90- 1.20)  0.75 (0.64- 0.88) | | Ref  0.62  <0.001 |

AOR= adjusted odds ratio, CI= confidence interval, WBJ= West Bank and Jerusalem.
* Adjusted for age-group, gender, educational level, occupation, monthly income, having a chronic disease, following a vegetarian diet, knowing someone with cancer, marital status, residency, and site of data collection.

Supplementary table 4: Bivariable logistic regression analyzing the association between recognizing colorectal cancer symptoms with mass/blood and participant characteristics.

COR= crude odds ratio, CI= confidence interval, WBJ= West Bank and Jerusalem.

| **Characteristic** | **Lump in the abdomen** | | **Blood in the stools** | | **Bleeding from back passage** | |
| --- | --- | --- | --- | --- | --- | --- |
|  | **COR (95% CI)** | **p-value** | **COR (95% CI)** | **p-value** | **COR (95% CI)** | **p-value** |
| **Age group**  18 to 44  45 or older | Ref  0.92 (0.79- 1.07) | Ref  0.29 | Ref  1.06 (0.91-1.23) | Ref  0.44 | Ref  1.07 (0.93-1.23) | Ref  0.36 |
| **Gender**  Female  Male | Ref  0.83 (0.72- 0.94) | Ref  0.005 | Ref  0.93 (0.82-1.05) | Ref  0.23 | Ref  0.81 (0.72- 0.91) | Ref  0.001 |
| **Educational level**  Illiterate  Primary  Preparatory  Secondary  Diploma  Bachelor  Postgraduate | Ref  1.83 (1.01- 3.32)  1.48 (0.86- 2.53)  1.87 (1.11- 3.14)  1.88 (1.09- 3.26)  1.57 (0.94- 2.63)  1.38 (0.74- 2.58) | Ref  0.048  0.15  0.018  0.023  0.08  0.31 | Ref  1.60 (0.90-2.83)  1.11 (0.66-1.88)  1.49 (0.90- 2.47)  1.66 (0.98- 2.82)  1.78 (1.08- 2.93)  2.23 (1.19- 4.15) | Ref  0.11  0.69  0.12  0.06  0.025  0.012 | Ref  1.42 (0.81-2.49)  1.10 (0.66-1.84)  1.29 (0.78- 2.12)  1.50 (0.89- 2.53)  1.44 (0.87- 2.36)  1.38 (0.76- 2.49) | Ref  0.22  0.72  0.32  0.13  0.15  0.29 |
| **Occupation**  Unemployed  Employed  Retired  Student | Ref  0.80 (0.69-0.92)  0.90 (0.56-1.44)  0.63 (0.51-0.77) | Ref  0.002  0.66  <0.001 | Ref  1.03 (0.91-1.18)  0.92 (0.60-1.42)  0.90 (0.74-1.09) | Ref  0.62  0.70  0.29 | Ref  0.94 (0.83-1.07)  0.78 (0.51-1.17)  0.70 (0.58-0.85) | Ref  0.34  0.23  <0.001 |
| **Monthly income**  < 1450 NIS  ≥ 1450 NIS | Ref  0.86 (0.75-0.99) | Ref  0.042 | Ref  1.28 (1.13-1.46) | Ref  <0.001 | Ref  0.92 (0.81-1.04) | Ref  0.19 |
| **Having a chronic disease**  No  Yes | Ref  0.83 (0.71-0.98) | Ref  0.026 | Ref  1.06 (0.91-1.24) | Ref  0.44 | Ref  1.09 (0.94- 1.26) | Ref  0.28 |
| **Following a vegetarian diet**  No  Yes | Ref  1.54 (1.24-1.92) | Ref  <0.001 | Ref  1.11 (0.92-1.35) | Ref  0.28 | Ref  1.57 (1.30-1.90) | Ref  <0.001 |
| **Knowing someone with cancer**  No  Yes | Ref  1.22 (1.07-1.39) | Ref  0.003 | Ref  1.04 (0.92-1.17) | Ref  0.57 | Ref  0.97 (0.86-1.09) | Ref  0.58 |
| **Marital status**  Single  Married  Divorced  Widowed | Ref  1.36 (1.18-1.56)  1.50 (0.74-3.07)  1.01 (0.64-1.58) | Ref  <0.001  0.26  0.97 | Ref  1.15 (1.01-1.31)  0.96 (0.52-1.78)  1.03 (0.66-1.58) | Ref  0.04  0.89  0.91 | Ref  1.35 (1.19-1.53)  1.37 (0.74-2.52)  0.96 (0.63-1.45) | Ref  <0.001  0.32  0.84 |
| **Residency**  Gaza Strip  WBJ | Ref  0.68 (0.59-0.78) | Ref  <0.001 | Ref  1.09 (0.97-1.24) | Ref  0.15 | Ref  0.76 (0.67-0.85) | Ref  <0.001 |
| **Site of data collection**  Public spaces  Hospitals  Primary healthcare centers | Ref  0.93 (0.79-1.09)  1.06 (0.90-1.25) | Ref  0.38  0.49 | Ref  1.11 (0.95-1.29)  0.89 (0.76-1.04) | Ref  0.18  0.14 | Ref  1.14 (0.99-1.32)  0.91 (0.79-1.06) | Ref  0.07  0.23 |

| **Characteristic**  Table 3: Association between recognizing breast symptoms and participant characteristics.  Table 3: Association between recognizing breast symptoms and sociodemographic factors. | **Unexplained weight loss** | | **Unexplained generalized fatigue** | | **Unexplained loss of appetite** | | **Anemia** | |
| --- | --- | --- | --- | --- | --- | --- | --- | --- |
|  | **COR (95% CI)** | **p-value** | **COR (95% CI)** | **p-value** | **COR (95% CI)** | **p-value** | **COR (95% CI)** | **p-value** |
| **Age group**  18 to 44  45 or older | Ref  1.15 (0.98-1.34) | Ref  0.08 | Ref  1.02 (0.88-1.19) | Ref  0.76 | Ref  0.96 (0.83-1.11) | Ref  0.58 | Ref  1.50 (1.30-1.74) | Ref  <0.001 |
| **Gender**  Female  Male | Ref  0.75 (0.66-0.86) | Ref  <0.001 | Ref  0.79 (0.70-0.90) | Ref  <0.001 | Ref  0.76 (0.67-0.85) | Ref  <0.001 | Ref  1.08 (0.96-1.22) | Ref  0.20 |
| **Educational level**  Illiterate  Primary  Preparatory  Secondary  Diploma  Bachelor  Postgraduate | Ref  1.79 (0.97- 3.29)  1.20 (0.70- 2.07)  1.29 (0.76- 2.18)  1.45 (0.83- 2.52)  1.21 (0.72- 2.05)  1.21 (0.64- 2.27) | Ref  0.06  0.51  0.35  0.19  0.47  0.55 | Ref  1.27 (0.70- 2.30)  1.11 (0.64- 1.91)  1.14 (0.67- 1.92)  1.15 (0.66- 1.99)  1.23 (0.73- 2.08)  1.35 (0.71- 2.54) | Ref  0.43  0.71  0.63  0.63  0.43  0.36 | Ref  0.92 (0.51- 1.66)  0.83 (0.49- 1.44)  0.84 (0.50- 1.42)  0.81 (0.47- 1.40)  0.82 (0.49- 1.39)  0.96 (0.52- 1.79) | Ref  0.79  0.52  0.52  0.45  0.47  0.90 | Ref  2.08 (1.15- 3.76)  1.11 (0.66- 1.89)  0.96 (0.58- 1.60)  0.91 (0.53- 1.54)  1.01 (0.61- 1.68)  0.89 (0.49- 1.63) | Ref  0.015  0.69  0.88  0.72  0.96  0.71 |
| **Occupation**  Unemployed  Employed  Retired  Student | Ref  0.86 (0.75-0.99)  0.83 (0.53-1.30)  0.71 (0.58-0.87) | Ref  0.030  0.42  0.001 | Ref  1.00 (0.87-1.15)  0.80 (0.52-1.23)  0.77 (0.63-0.94) | Ref  0.97  0.30  0.009 | Ref  0.80 (0.70-0.91)  0.70 (0.47-1.07)  0.68 (0.56-0.82) | Ref  0.001  0.10  <0.001 | Ref  1.12 (0.98-1.27)  1.00 (0.66-1.53)  0.74 (0.62-0.90) | Ref  0.10  0.99  0.002 |
| **Monthly income**  < 1450 NIS  ≥ 1450 NIS | Ref  0.95 (0.83-1.08) | Ref  0.44 | Ref  1.13 (0.99-1.29) | Ref  0.07 | Ref  0.96 (0.84-1.08) | Ref  0.48 | Ref  1.17 (1.03-1.32) | Ref  0.015 |
| **Having a chronic disease**  No  Yes | Ref  1.08 (0.92-1.27) | Ref  0.33 | Ref  1.00 (0.85-1.17) | Ref  0.99 | Ref  0.98 (0.84- 1.14) | Ref  0.77 | Ref  1.25 (1.08-1.46) | Ref  0.003 |
| **Following a vegetarian diet**  No  Yes | Ref  0.71 (0.59-0.85) | Ref  <0.001 | Ref  0.51 (0.43-0.62) | Ref  <0.001 | Ref  0.66 (0.56-0.79) | Ref  <0.001 | Ref  0.51 (0.43-0.61) | Ref  <0.001 |
| **Knowing someone with cancer**  No  Yes | Ref  1.64 (1.45-1.87) | Ref  <0.001 | Ref  1.64 (1.44-1.86) | Ref  <0.001 | Ref  1.54 (1.37-1.74) | Ref  <0.001 | Ref  1.36 (1.21-1.53) | Ref  <0.001 |
| **Marital status**  Single  Married  Divorced  Widowed | Ref  1.34 (1.17-1.53)  1.09 (0.57-2.07)  1.78 (1.08-2.92) | Ref  <0.001  0.80  0.023 | Ref  1.15 (1.00-1.31)  0.65 (0.35-1.18)  1.11 (0.71-1.74) | Ref  0.050  0.16  0.65 | Ref  1.15 (1.01-1.31)  0.64 (0.35-1.16)  1.08 (0.71-1.65) | Ref  0.032  0.14  0.71 | Ref  1.26 (1.11-1.44)  1.27 (0.69-2.34)  1.49 (0.97-2.30) | Ref  <0.001  0.45  0.07 |
| **Residency**  Gaza Strip  WBJ | Ref  0.93 (0.81-1.05) | Ref  0.25 | Ref  1.08 (0.95-1.23) | Ref  0.23 | Ref  0.87 (0.77-0.98) | Ref  0.022 | Ref  1.17 (1.04-1.32) | Ref  0.010 |
| **Site of data collection**  Public spaces  Hospitals  Primary healthcare centers | Ref  1.30 (1.11-1.52)  1.30 (1.12-1.53) | Ref  0.001  0.001 | Ref  1.20 (1.03-1.39)  1.12 (0.96-1.31) | Ref  0.022  0.14 | Ref  1.39 (1.20-1.60)  1.13 (0.98-1.31) | Ref  <0.001  0.10 | Ref  1.88 (1.62-2.17)  1.18 (1.02-1.36) | Ref  <0.001  0.026 |

Supplementary table 5: Bivariable logistic regression analyzing the association between recognizing colorectal cancer symptoms of a non-specific nature and participant characteristics.

COR= crude odds ratio, CI= confidence interval, WBJ= West Bank and Jerusalem.

| **Characteristic**  Supplementary table 6: Bivariable logistic regression analyzing the association between recognizing other gastrointestinal symptoms and participant characteristics. | **Feeling persistently full** | | **Change in bowel habits** | | | **Persistent pain in the abdomen** | | **Bowel does not completely empty** | | **Pain in the back passage** | |
| --- | --- | --- | --- | --- | --- | --- | --- | --- | --- | --- | --- |
|  | **COR (95% CI)** | **p-value** | **COR (95% CI)** | **p-value** | **COR (95% CI)** | | **p-value** | **COR (95% CI)** | **p-value** | **COR (95% CI)** | **p-value** |
| **Age group**  18 to 44  45 or older | Ref  1.02 (0.89-1.18) | Ref  0.74 | Ref  1.36 (1.18-1.57) | Ref  <0.001 | Ref  0.88 (0.76-1.01) | | Ref  0.07 | Ref  1.21 (1.06-1.40) | Ref  0.007 | Ref  1.19 (1.04-1.37) | Ref  0.012 |
| **Gender**  Female  Male | Ref  0.85 (0.76-0.96) | Ref  0.010 | Ref  0.97 (0.86-1.09) | Ref  0.60 | Ref  1.07 (0.95-1.20) | | Ref  0.27 | Ref  0.95 (0.85-1.07) | Ref  0.40 | Ref  1.00 (0.89-1.12) | Ref  0.99 |
| **Educational level**  Illiterate  Primary  Preparatory  Secondary  Diploma  Bachelor  Postgraduate | Ref  1.68 (0.95-2.95)  1.52 (0.91-2.55)  1.32 (0.80-2.18)  1.22 (0.72-2.05)  1.50 (0.91-2.46)  1.65 (0.91-3.00) | Ref  0.07  0.11  0.27  0.46  0.11  0.10 | Ref  1.52 (0.86-2.68)  0.98 (0.59-1.65)  1.03 (0.62-1.69)  1.03 (0.61-1.74)  1.23 (0.75-2.02)  1.14 (0.63-2.08) | Ref  0.15  0.95  0.92  0.92  0.42  0.66 | Ref  1.40 (0.80-2.45)  1.23 (0.73-2.06)  1.53 (0.93-2.53)  1.86 (1.10-3.14)  2.03 (1.23-3.34)  2.48 (1.36-4.54) | | Ref  0.24  0.44  0.09  0.02  0.005  0.003 | Ref  1.42 (0.81-2.49)  0.97 (0.58-1.63)  1.02 (0.62-1.68)  0.95 (0.57-1.60)  1.09 (0.66-1.78)  1.09 (0.60-1.97) | Ref  0.22  0.92  0.93  0.86  0.74  0.78 | Ref  1.23 (0.70-2.16)  0.91 (0.54-1.52)  0.94 (0.57-1.55)  0.87 (0.52-1.46)  0.95 (0.58-1.56)  1.26 (0.70-2.28) | Ref  0.46  0.72  0.82  0.59  0.84  0.44 |
| **Occupation**  Unemployed  Employed  Retired  Student | Ref  1.01 (0.89-1.15)  0.63 (0.42-0.96)  0.78 (0.65-0.94) | Ref  0.82  0.030  0.009 | Ref  1.13 (0.99-1.28)  1.34 (0.88-2.05)  1.09 (0.90-1.31) | Ref  0.06  0.18  0.39 | Ref  1.16 (1.02-1.32)  1.10 (0.73-1.67)  1.11 (0.92-1.34) | | Ref  0.02  0.65  0.28 | Ref  1.07 (0.95-1.21)  0.93 (0.62-1.40)  0.85 (0.71-1.03) | Ref  0.28  0.73  0.10 | Ref  1.05 (0.93-1.19)  1.04 (0.69-1.57)  0.86 (0.71-1.03) | Ref  0.42  0.85  0.10 |
| **Monthly income**  < 1450 NIS  ≥ 1450 NIS | Ref  1.15 (1.01-1.30) | Ref  0.031 | Ref  1.40 (1.24-1.58) | Ref  <0.001 | Ref  1.44 (1.27-1.62) | | Ref  <0.001 | Ref  1.45 (1.28-1.64) | Ref  <0.001 | Ref  1.29 (1.14-1.45) | Ref  <0.001 |
| **Having a chronic disease**  No  Yes | Ref  1.01 (0.87-1.17) | Ref  0.93 | Ref  1.06 (0.92-1.23) | Ref  0.43 | Ref  0.97 (0.84-1.13) | | Ref  0.71 | Ref  1.07 (0.92-1.23) | Ref  0.38 | Ref  1.11 (0.96-1.29) | Ref  0.15 |
| **Following a vegetarian diet**  No  Yes | Ref  0.40 (0.33-0.48) | Ref  <0.001 | Ref  0.40 (0.33-0.48) | Ref  <0.001 | Ref  0.73 (0.61-0.87) | | Ref  <0.001 | Ref  0.43 (0.36-0.52) | Ref  <0.001 | Ref  0.52 (0.43-0.63) | Ref  <0.001 |
| **Knowing someone with cancer**  No  Yes | Ref  1.36 (1.21-1.53) | Ref  <0.001 | Ref  1.30 (1.16-1.46) | Ref  <0.001 | Ref  1.16 (1.03-1.30) | | Ref  0.015 | Ref  1.32 (1.17-1.48) | Ref  <0.001 | Ref  1.22 (1.09-1.37) | Ref  0.001 |
| **Marital status**  Single  Married  Divorced  Widowed | Ref  1.20 (1.06-1.36)  1.28 (0.69-2.36)  1.11 (0.73-1.68) | Ref  0.005  0.43  0.64 | Ref  1.02 (0.90-1.16)  0.77 (0.42-1.39)  1.64 (1.05-2.56) | Ref  0.79  0.39  0.028 | Ref  0.90 (0.79-1.02)  0.64 (0.36-1.17)  0.88 (0.58-1.34) | | Ref  0.10  0.15  0.55 | Ref  1.17 (1.03-1.32)  0.82 (0.45-1.49)  1.13 (0.75-1.71) | Ref  0.017  0.51  0.55 | Ref  1.03 (0.91-1.17)  0.88 (0.49-1.61)  1.17 (0.78-1.77) | Ref  0.65  0.68  0.44 |
| **Residency**  Gaza Strip  WBJ | Ref  1.16 (1.03-1.30) | Ref  0.017 | Ref  1.46 (1.30-1.65) | Ref  <0.001 | Ref  1.29 (1.15-1.45) | | Ref  <0.001 | Ref  1.40 (1.24-1.57) | Ref  <0.001 | Ref  1.24 (1.10-1.39) | Ref  <0.001 |
| **Site of data collection**  Public spaces  Hospitals  Primary healthcare centers | Ref  1.55 (1.35-1.80)  1.14 (0.99-1.32) | Ref  <0.001  0.07 | Ref  1.24 (1.07-1.43)  0.85 (0.73-0.98) | Ref  0.003  0.025 | Ref  1.20 (1.04-1.39)  0.79 (0.69-0.92) | | Ref  0.013  0.002 | Ref  1.36 (1.18-1.57)  0.84 (0.73-0.97) | Ref  <0.001  0.019 | Ref  1.05 (0.91-1.21)  0.77 (0.67-0.89) | Ref  0.46  <0.001 |

COR= adjusted odds ratio, CI= confidence interval, WBJ= West Bank and Jerusalem.
